# Evaluation of Low Temperature and Salinity as a Treatment of Atlantic Salmon against Amoebic Gill Disease

**DOI:** 10.3390/microorganisms10020202

**Published:** 2022-01-18

**Authors:** Jemma Hudson, Mark Adams, Khattapan Jantawongsri, Tim Dempster, Barbara F. Nowak

**Affiliations:** 1 Institute for Marine and Antarctic Studies (IMAS), University of Tasmania, Launceston, TAS 7250, Australia; jhudson6@utas.edu.au (J.H.); Mark.Adams@utas.edu.au (M.A.); Khattapan.Jantawongsri@utas.edu.au (K.J.); 2 Sustainable Aquaculture Laboratory—Temperate and Tropical (SALTT), School of BioSciences, Faculty of Science, University of Melbourne, Melbourne, VIC 3010, Australia; dempster@unimelb.edu.au

**Keywords:** mariculture, salmon, *Neoparamoeba perurans*

## Abstract

Amoebic gill disease (AGD) is a significant health issue for Atlantic salmon farmed in a marine environment. While the disease is currently managed using freshwater or hydrogen peroxide baths, there is a need to develop other treatments. The aims of this study were to examine the effect of salinity (0 ppt and 35 ppt) and temperature (3 °C and 15 °C) on attachment and survival of *Neoparamoeba perurans* in vitro over short exposure times (15 min and 2 h) and to assess the efficacy of reduced temperature (3 °C) as treatment for Atlantic salmon affected by AGD. In vitro freshwater 3 °C was at least as effective as freshwater 15 °C and the attachment was significantly lower after 2 h in freshwater 3 °C than freshwater 15 °C. In vivo there was no difference between the fish treated with freshwater 15 °C for 2 h or freshwater 3 °C. This study showed that despite exposure to low temperature reducing attachment of *N. perurans* to their substrate in vitro, 15 min cold-water bath treatment was not more effective at reducing AGD in Atlantic salmon than current commercial 2 h freshwater bath.

## 1. Introduction

Amoebic gill disease (AGD) was first reported in the late 1980s, in Atlantic salmon smolt farmed at Bruny Island in Tasmania, Australia, and in coho salmon (*Oncorhynchus kisutch*) farmed in Washington, USA [1,2]. Since then, AGD outbreaks have been seen across finfish mariculture worldwide. AGD is caused by *Neoparamoeba perurans*, which can parasitise gills of a number of host species [3]. The impact of the disease has been substantial to the salmon industry, causing significant increases in production costs due to treatment, mortalities, and reduced growth of fish. Treatment is estimated to cost the Atlantic salmon industry in Tasmania around $40 million annually, increasing the cost of production by $1.50/kg [4]. This amounts to a significant proportion of costs in an industry that had a production value of $851 million in 2018−2019 [5]. Comparisons of impacts of parasitic diseases on mariculture industry globally have suggested that AGD has one of the highest economic impacts [6]. The methods to treat fish affected by AGD on a commercial scale are limited to the use of freshwater or hydrogen peroxide baths.

Alternative treatment methods utilise the environmental limitations of pathogens. When the optimal environmental conditions of the host and pathogen are understood, they can be manipulated in a way that limits the survival of the pathogen without harming the host. Environmental conditions that can be manipulated include salinity and temperature. For example, freshwater bathing has been used to treat AGD or sea lice (*Lepeophtheirus salmonis*) affected Atlantic salmon, and the addition of salt or a temporary increase in temperature have been used to treat silver perch (*Bidyanus bidyanus*) affected by ichthyophthiriasis [7,8]. 

Low temperature treatments have also been examined for Atlantic salmon affected by sea lice. The sea lice require temperatures greater than 4 °C to complete their lifecycle, although the lower limits of their thermal range are not known [9]. Bath treatments of −1 °C seawater reduced the load of mobile sea lice on the fish; however, there were some negative effects on the salmon, with fin and skin damage when the treatment was longer than 30 min [10]. Treatments that lasted under 10 min reduced sea lice load with minimum or no negative effects on salmon, though overall cold-water treatments were less effective at sessile lice removal than freshwater treatments [10].

The causative agent of AGD, *N. perurans*, is an amphizoic amoebae approximately 20–30 µm in diameter found in temperate marine environments [11,12,13]. The effects of temperature on *N. perurans* have been studied In vitro. Optimal survival and growth were observed in temperatures of 15 °C or above and salinities of 35 ppt [14,15]. The combination of these conditions allowed for *N. perurans* populations to double every 14 h [15]. Lower temperatures, for example, 4 °C, resulted in slower growth and reduced attachment [14,15,16]. Although freshwater bath has been the most common treatment against AGD, the potential for the use of low temperature in AGD management has not been evaluated.

The aims of this study were to examine the effect of salinity and temperature on attachment and survival of *N. perurans* In vitro over short exposure times and to assess the efficacy of reduced temperature as treatment for Atlantic salmon affected by AGD.

## 2. Materials and Methods

### 2.1. Ethics

The experiment was done with approval from the Animal Ethics Committee University of Tasmania (Ethics number A0017812) granted on 21 December 2018. 

### 2.2. Experimental Treatments

For both In vitro and In vivo experiments, four treatments were examined, combining high and low temperatures and high and low salinities. An ambient temperature of 15 °C was chosen, and a seawater 15 °C (SW 15 °C) treatment used as a negative control. A freshwater 15 °C (FW 15 °C) treatment was used as the positive control treatment. Two cold-water treatments were examined, both freshwater (FW 3 °C) and seawater (SW 3 °C) at 3 °C. 

### 2.3. Isolation of N. perurans

The amoebae were isolated from fish from an infection tank that was also the source of amoebae for In vivo experiments in this study. The infection tank with AGD affected rainbow trout and Atlantic salmon has been maintained at the Aquaculture Research and Teaching Facility Centre IMAS (Launceston, Tasmania) and was used to provide amoebae for the In vitro and In vivo experiments, following previously published methods [17]. Amoebae used for the infection challenge were isolated from the gills of AGD affected Atlantic salmon as previously described [17,18]. The amoebae were left at 15 °C to allow them to multiply overnight. The following day the supernatant was poured off and petri dishes rinsed. Once clean, 5 mL of freshwater was added to each petri dish to detach the amoebae. The isolated amoebae were collected in a 1 L shott bottle. The amoebae number was estimated by counting the number of amoebae in 50 × 10 μL droplets on microscope slides and multiplying the average by the overall volume of the water. 

### 2.4. In Vitro Experiment

A trial was run to examine the effects of cold freshwater 0 ppt (FW) and seawater 35 ppt (SW) on the attachment and survival of *N. perurans*. 

Ten replicate wells in 48 well microliter plates were used for each treatment. Seawater suspended isolate (0.5 mL) with a known concentration of amoebae was pipetted into each well for each plate to result in 750 amoebae/well. The plates were then left for 30 min to allow time for the amoebae to attach. The water was removed from each well and 0.5 mL of the treatment water (FW 15 °C, FW 3 °C, SW 15 °C, or SW 3 °C) was added to each well. Each plate was placed in an incubator set to the temperature of the respective treatment. 

The amoebae were exposed for either 15 min or 2 h to the treatment. At the end of the treatment the plate was taken out and observed under an inverted dissection microscope (Olympus Ck2, 10×, Marshall Scientific, Hampton, NH, USA). To observe attachment of the amoebae, five photos were taken from each well in a transect from the left to the right of the each well. For each field of view, counts were done of the attached and detached amoebae. Attachment was assessed based on morphology and movement of the amoebae. As photos were taken, the plates were gently shaken, and the number of fully detached amoebae noted. Total number of amoebae was used to calculate the percentage of the attached amoebae. 

Once post-treatment photos were taken, all treatments were brought back to 15 °C seawater by adding 0.6 mL of 25 °C 70 ppt seawater to the FW 3 °C treatment wells, 0.6 mL of 25 °C 35 ppt seawater to the SW 3 °C treatment wells, 0.6 mL of 15 °C 70 ppt seawater to the FW 15 °C treatment wells, and 0.6 mL of 15 °C 35 ppt seawater to the SW 15 °C treatment wells. The plates were left for amoebae to settle for 30 min before 0.6 mL of water was removed from each well. A 0.5 mL neutral red solution was added to each well and the plates left at room temperature for 30 min for the amoebae to absorb the stain. The plates were then observed under the microscope to determine survival of the amoebae.

### 2.5. In Vivo Experiment

#### 2.5.1. Salmon Husbandry

One hundred and fifty (150) Atlantic salmon smolt (all female diploid stock ~ 620.05 ± 164.83 g) were transferred from a commercial hatchery and housed at the University of Tasmania, Newnham campus in a freshwater 3500 L recirculating system until the start of the trial. The salmon were acclimated to 35 ppt seawater over a period of 2 months, prior to the infection challenge, starting at 10 ppt and raised to 30 ppt over 3 weeks, then held at this salinity for 5 weeks with the increase to 35 ppt at the beginning of the trial. After the challenge, the fish were maintained in the challenge tank until treatment. A heater/chiller unit was used to maintain constant temperature in the system. The challenge tank was monitored daily for changes in water quality, with parameters being kept as: nitrite < 2–5 mg/L, nitrate < 160 mg/L, ammonia < 2 mg/L, pH = 7.8 ± 0.2, dissolved oxygen >80% saturation, temperature 16 ± 1 °C (post acclimation), and salinity = 35 ppt (post acclimation). To maintain water quality, water changes were done twice weekly, exchanging 1/3 of the water in the tank. Fish were fed twice daily with a pelleted (0.4 mL) Skretting feed at approximately 0.75%/body weight. Fish were not fed 24 h prior to challenge or to sampling.

#### 2.5.2. AGD Challenge

For infection with AGD, the salmon were exposed to total of 1423 amoebae/L over 4 weeks; the individual exposures were done using methods modified from Morrison et al. [17]. Due to the low numbers of amoebae available, infection was done in 5 rounds over 4 weeks; at each time point between 147 and 507 amoebae/L (average 284.6 amoebae/L) were added. For each round of exposure to amoebae, the water in the tank was dropped to 2000 L and the heater/chiller unit, foam fractionator, bead filter, UV, and tank pump were turned off. A watering can was filled 4/5 with water from the tank and the amoebae isolate mixed with the water. The solution was then poured evenly across the infection tank. After 1–2 h (depending on infection round), the tank pump, heater/chiller unit, and bead filter were turned on again, and after 24 h the foam fractionator and UV were turned on again. Each week post infection, 10 fish were checked for AGD by taking a gross gill score as described previously [19], until there was an average gill score of 2.

#### 2.5.3. Experimental Set-Up

Twelve fish were netted from the challenge tank, to measure infection and disease levels pre-treatment. Treatment was done 24 h after the initial sampling. The remaining 112 fish from the infection tank were divided into 26−28 fish per treatment and transferred into holding tanks (850 L) with either freshwater or seawater at 3 °C or 15 °C. Fish were treated in FW at 15 °C treatment for 2 h to simulate industry standard FW treatment, whereas all other treatments were conducted for 15 min. Post treatment, between 9 and 11 fish were sampled from each treatment. After treatment and initial post-treatment sampling, the remaining fish were transferred to a 5000 L seawater RAS (35 ppt, 15 °C) with biological and two UV filters, for one week. In this system, fish were divided by treatment into 12 tanks (triplicate tanks for each treatment) with 6 fish assigned to each tank. Individual tanks were surrounded by a plastic sheet, 1 m high from the top of the tank, to prevent spread of infection between tanks. Water quality was monitored daily in this system as done for the previous system, with water exchanged at approximately 10% per day via drum filter back-flushing. The fish were held for 1 week before final sampling.

The temperature and dissolved oxygen levels of treatment tanks were monitored immediately before and after treatment. Water quality was monitored in all tanks (water temperature 15–16 °C, D.O. saturation > 90%, pH 7.8–8.0, TA-N < 1 mg/L, NO_2_ < 0.25 mg/L, and NO_3_ < 10 mg/L).

#### 2.5.4. Sampling

For each sampling event, fish were euthanised using an anaesthetic bath (640 μg/L Aqui-S). Three methods were used to assess AGD. A gross score of AGD was taken as described previously [19]. The third right gill arch was swabbed using a cotton swab. The head of the swab was snapped off and placed in a 2 mL tube containing RNAlater^®^ (SigmaAldrich, Saint Louis, MO, USA) and stored on ice during sampling for *N. perurans* detection by quantitative PCR (qPCR). To investigate histopathology the whole gill basket was dissected out and left gill arches were placed into 120 mL jars filled with 90 mL Davidson’s fixative. 

#### 2.5.5. *N. perurans* Detection

Gill swabs were stored in the −80 °C freezer until DNA extraction followed by qPCR analysis to measure the load of *N. perurans* on the gills. For DNA extraction, swabs were shaken using a FastPrep-24TM 5G lysis system (MP Biomedicals) at 6 m/s for 40 sec. The samples were then transferred to a clean tube and spun down for 10 min at 14,000 rpm to form a pellet. DNA was extracted using the DNeasy Blood and Tissue kit (Qiagen, VIC, Australia) as per manufacturer’s protocol. Samples were normalised to 30 ng/µL. The qPCR assays were run in a ViiATM 7 system (Applied Biosystems, Thermo Fisher Scientific, VIC, Australia) in 384 well plates. 

The qPCR assay targeted the 18S rRNA gene of *N. perurans* (PERU) and the endogenous control gene (ELF) using previously described methods [20,21]. A plasmid standard curve of *N. perurans* and NTC (no template control) was run on each qPCR plate. Assays were done in triplicate for PERU and in duplicate for ELF and NTC. Quantity of *N. perurans* was calculated based on 30 ng of template DNA. 

#### 2.5.6. Histology

After 48 h, the Davidson’s fixative was replaced with 60 mL of 70% ethanol for storage until the samples were processed. The third left gill arch was removed from each sample and placed in a cassette for processing. Samples were trimmed, processed via standard protocol for histology, embedded in paraffin wax, sectioned at 5 μm through the posterior side of the gill and stained with haematoxylin and eosin (H&E). After staining, the sections were observed under a microscope (Olympus BH2, Marshall Scientific Hampton, Hampton, NH, USA, using objectives 4×−100×) and the percentage of gill filaments with at least one lesion, the percentage of gill filaments with amoebae positive lesions (at least one amoeba present on a lesion in the gill filament), and the percentage of all lesions with amoebae present were determined. As some filaments were not sectioned at the correct angle, a filament was counted only if >90% (estimated visually) of the filament was properly oriented. In cases where 40−60% of the gill was well oriented, the area that was correctly oriented was counted as a half filament. A gill filament was considered to be well-oriented if the lamellae were of equal length on either side of the central venous sinus.

#### 2.5.7. Statistical Analysis

The data from this experiment, including percentage of attachment and survival of the amoebae, gross gill score, and percentage of gill filaments with lesions, were analysed using *R* Version 4.1.1 [22]. All data collected were tested for normality using a Shapiro–Wilk test. As all data sets had non-normal distribution, the Kruskal–Wallis test was used to analyse the effect of treatments. All outliers were excluded from the analyses. If a significant difference was observed, a post-hoc pairwise Wilcoxon test was run to identify which treatments were significantly different from each other with *p* < 0.05 were considered statistically significant. Results were presented as boxplots with median and inter-quartile range.

## 3. Results

### 3.1. In Vitro Experiment

**Figure 1:**
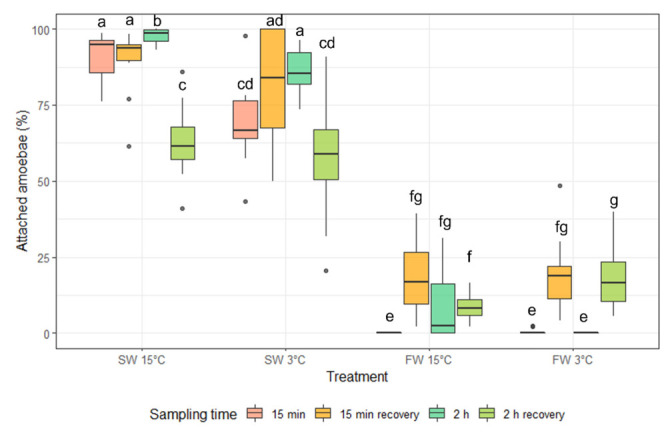
The percentage of *N. perurans* attached to well plates (median and interquartile range) at 15 min and 2 h exposure to treatment conditions, and after 24 h recovery for each exposure. Dots indicate outliers (not included in the analysis). Different letters indicate significant difference between groups (*p* < 0.05).

The percentage of attached amoebae at 15 min in treatment conditions decreased significantly for the FW 3 °C and FW 15 °C compared to both seawater treatments (Figure 1). There was no significant difference between the two freshwater treatments at 15 min; however, there was a significant difference between these treatments after 2 h with significantly higher percentage of attached amoebae at FW 15 °C (Figure 1). At 15 min and 2 h the percentage of attached amoebae was significantly lower in the SW 3 °C compared to the SW 15 °C. When allowed 24 h to recover, amoebae exposed for 15 min had increased attachment for FW 3 °C and FW 15 °C; however, there were still significantly lower than after both SW treatments. For amoebae exposed to treatment conditions for 2 h, there was a significant increase in attachment after recovery for the FW 3 °C treatment, which was significantly different from FW 15 °C post-recovery. There was a decrease in attachment after recovery for both seawater treatments. However, the percentage of attached amoebae was significantly higher for these treatments than for the FW 3 °C and FW 15 °C treatments. The results suggested that In vitro FW 3 °C was at least as effective as FW 15°C and that the attachment was significantly lower after 2 h in FW 3 °C than FW 15 °C. 

**Figure 2:**
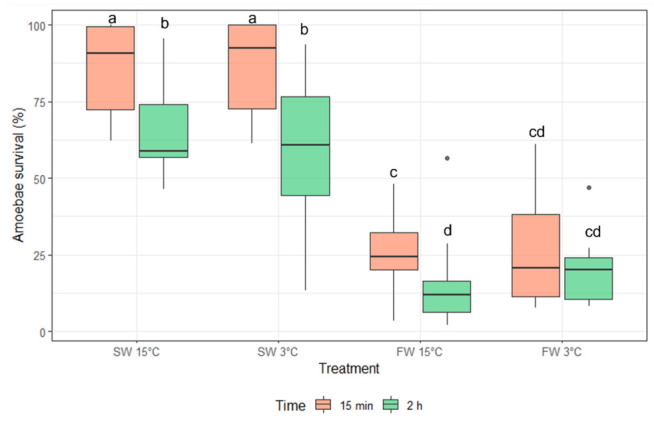
The survival (median and interquartile range) of *N. perurans* based on neutral red inclusion, 24 h after recovery for 15 min and 2 h treatments. Dots denote outliers (not included in the analysis). Different letters indicate significant difference between groups (*p* < 0.05).

The percentage of amoebae that survived after exposure was significantly lower for both freshwater treatments compared to both seawater treatments for both 15 min and 2 h (Figure 2). Although the percentage of amoebae that survived FW 3 °C did not significantly decrease with a greater exposure time of 2 h compared to 15 min, a reduction in the percentage of surviving amoebae after 2 h was seen for all other treatments (Figure 2). There was no significant difference between FW 3 °C and FW 15 °C. Overall survival at 2 h for the seawater treatment was significantly higher than in both freshwater conditions after either exposure time. There were still some amoebae alive after 2 h FW treatment regardless of temperature.

Morphological differences were observed but not quantified. Amoebae exposed to SW 3 °C detached but generally remained in subspherical form with pseudopodia still out. Amoebae exposed FW 3 °C were much more rounded than the amoebae exposed to seawater, but often not completely spherical as they were in FW 15 °C. 

### 3.2. In Vivo Experiment

#### 3.2.1. Gross Gill Score

**Figure 3:**
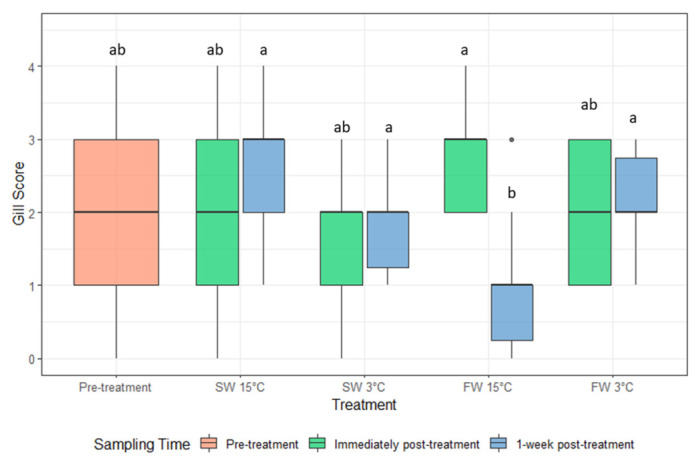
The gill score of AGD affected Atlantic salmon (median and interquartile range) pre-treatment, immediately post-treatment, and 1 week post-treatment. Dots indicate outliers. Different letters indicate significant difference between groups (*p* < 0.05).

The gross gill score for the fish given the FW 15 °C bath decreased from the immediately-post treatment samples to the one-week post-treatment samples (Figure 3). For the fish sampled one week post-treatment, fish given the FW 15 °C bath had lower gill scores compared to the FW 3 °C, SW 3 °C, and SW 15 °C treatment groups. There was no significant difference in gill score for any treatment group immediately post treatment or one-week post-treatment compared to the pre-treatment scores (Figure 3).

#### 3.2.2. Histology

**Figure 4:**
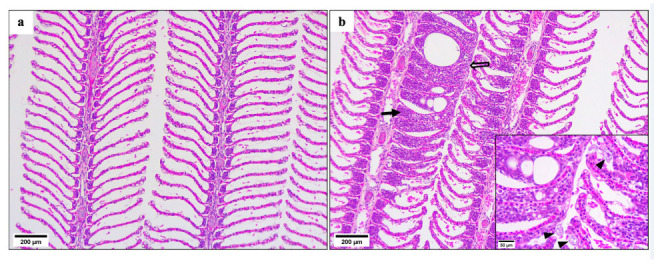
Histological sections of Atlantic salmon gills stained with H&E: (**a**) normal gills, (**b**) gill lesions with epithelial hyperplasia (line arrow) and lamellar fusion (block arrow), inset—*N. perurans* (arrowhead).

**Figure 5:**
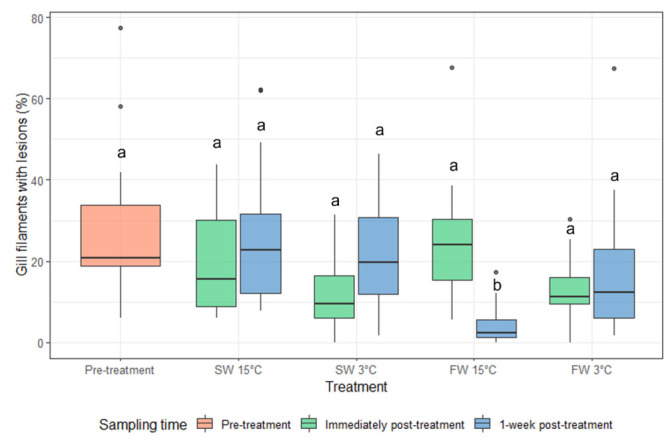
The percentage of gill filaments with lesions (median and interquartile range) pre-treatment, immediately post-treatment, and 1 week post-treatment. Dots indicate outliers (not included in the statistical analysis). Different letters indicate significant difference between groups (*p* < 0.05).

**Figure 6:**
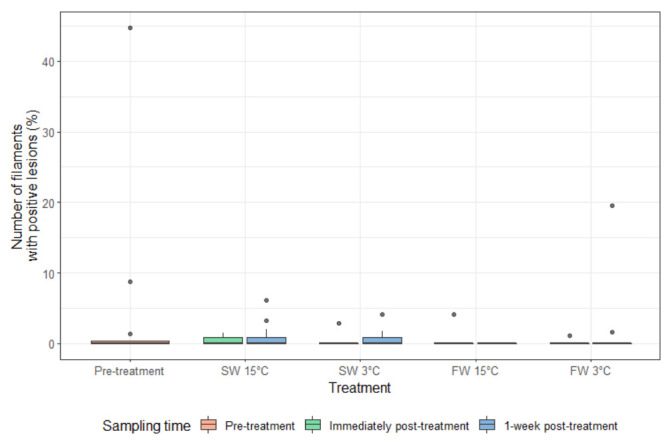
The percentage of filaments with lesions positive for amoebae (median and interquartile range) pre-treatment, immediately post-treatment, and 1 week post-treatment. Dots indicate outliers (not included in the analysis).

**Figure 7:**
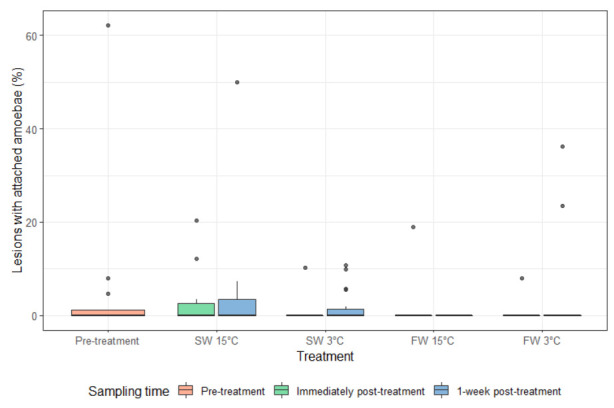
The percentage of lesions with amoebae (median and interquartile range) pre-treatment, immediately post-treatment, and 1 week post-treatment. Dots indicate outliers (not included in the analysis).

AGD lesions were present before treatment (Figure 4). A significant reduction in the percentage of filaments with lesions was observed for fish given the FW 15 °C treatment at one week post treatment compared to pre-treatment and immediately post-treatment fish as well as all other treatments (Figure 5). The presence of amoebae on the gills was highly variable and there was no significant difference for percentage of lesions positive for amoebae between any treatments and time post-treatment (Figure 6 and Figure 7).

#### 3.2.3. qPCR

**Figure 8:**
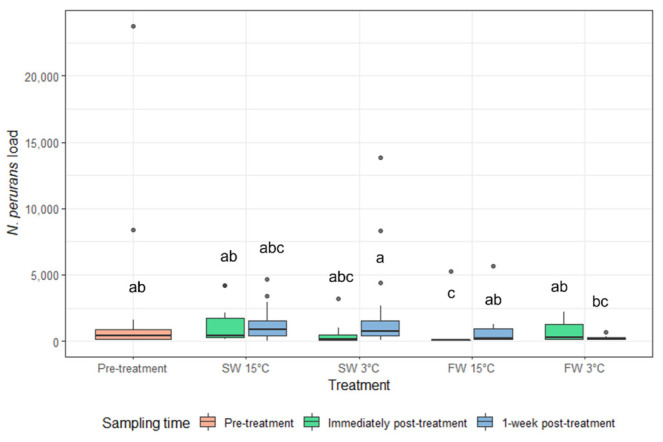
The load of *N. perurans* from gill swabs of Atlantic salmon (median and interquartile range) pre-treatment, immediately post-treatment, and 1 week post-treatment. Dots indicate outliers (not included in the analysis). Different letters represent significant difference between groups (*p* < 0.05).

The load of *N. perurans* was significantly reduced immediately post-treatment in FW 15 °C in comparison to pre-treatment load and it was significantly lower than SW 3 °C immediately post-treatment (Figure 8). There was no difference in amoebae load on the gills of the fish given other treatments at either sampling time, except one-week post-treatment FW 3 °C was lower than SW 3 °C. The load of *N. perurans* on the gills was highly variable, particularly pre-treatment (Figure 8).

## 4. Discussion

This study confirmed that exposure to low temperature reduced attachment of *N. perurans* In vitro even in short exposure times of 15 min. Amoebae exposed to 3 °C seawater had increased detachment at 15 min compared to 15 °C seawater, confirming that cold water acts as a stressor to *N. perurans* independently of salinity. However, exposure to 3 °C seawater did not reduce amoebae attachment as much as FW 15 °C, suggesting that temperature is less impactful as a stressor. FW 3 °C was equally or more effective In vitro as FW 15 °C with the greatest detachment seen after 2 h exposure in FW 3 °C. 

Recovery of amoebae, defined by attachment after 24 h in control conditions post-treatment, was significantly greater for FW 3 °C than FW 15 °C after 2 h treatment, whereas there was no significant difference in the recovery between the two temperatures after 15 min FW treatment. The overall level of survival across all treatments in this study was higher than previous findings, which suggested that *N. perurans* that became pseudocysts did not survive in freshwater for longer than 1 h In vitro [23]. However, high recovery rates after 1 h but reduced recovery at 2−3 h were observed in other studies [24,25]. This disparity in freshwater survival times could relate to a difference in the isolate of *N. perurans* used and the culture conditions, including the duration in culture. Overall, this study does corroborate that short 15 min exposures to freshwater allow high recovery rates of *N. perurans* [23,24,25]. 

Whereas the low temperature showed some promise In vitro, the In vivo experiment showed that 3 °C treatment for a duration of 15 min in either freshwater or seawater is unlikely to be an effective replacement for current commercial 2 h freshwater baths. 

There was no lasting reduction in gill lesions based on gill scores or histology for either cold-water treatments, in contrast to the 2 h freshwater treatment. Similarly, there was no decrease in *N. perurans* load on the gills of Atlantic salmon treated with cold water, unlike in the 2 h freshwater treatment where there was significant reduction immediately after treatment. 

The duration of treatment most likely had an impact on the efficacy for removing amoebae from the gills of fish. Freshwater bath treatments depend not only on the stress-response of amoebae to cause detachment from the gills, but on host-dependent mechanisms such as thinning and sloughing of the gill mucus, together with amoebae and cellular debris [23,26]. The mucus on the gills of fish is seawater-stable, and when exposed to freshwater is altered with increased hydration and decreased viscosity, resulting in a greater sloughing ability [27]. The time required for this process may be longer than 15 min, with studies showing mucus viscosity decreased in soft freshwater at 1 h and hard freshwater at 3 h [26]. Amoebae that detached may not have been completely removed from gills after 15 min and could have been able to recover once the fish were returned to seawater.

This disparity between In vitro and In vivo experiments has been seen previously for freshwater bathing at short durations. Despite detachment of *N. perurans* after 3 min exposures to freshwater In vitro, 3 min freshwater baths showed inconsistent reductions of amoebae from gills, even when repeated over multiple days [23]. Several factors, such as intercellular interactions, mucus, and development of lesions may help *N. perurans* to stay attached longer on the gills than they would on plastic substrates In vitro. The rate that *N. perurans* can be removed from the gills following any treatment is likely to be influenced by the hydration of mucus and subsequent reduction in viscosity as well as interactions between amoebae and the gill epithelium [28,29,30,31,32]. Gill mucus is suspected to aid in protecting amoebae from freshwater exposure in the short term, which may reduce detachment as well [23]. 

Water hardness may also be a factor in the efficacy of bath treatments, particularly involving freshwater. Soft freshwater is more effective at reducing AGD, as it causes an increase in the disintegration and shedding of hyperplastic tissue in gill lesions [31]. This is also likely to do with changes in the mucus layer, with mucus viscosity decreasing faster in soft freshwater than hardwater as the disentanglement of mucin fibres is sped up in soft water [26]. 

In both 3 °C treatments, the fish activity was reduced compared to the 15 °C treatments. This may have had an impact on the respiratory ability of the fish, if there was reduced opercular movement. In turn, the fish may not be getting as much water through the gills to hydrate mucus or remove amoebae. The size of fish may also be a factor that could affect efficacy of cold-water treatment. Larger fish have a greater thermal inertia, and therefore may be less affected by cold water. 

In this study, the gross gill scores and the percentage of filaments with lesions in histology showed the same results, as is observed in more severe cases of AGD [28]. Although the use of gill scores to estimate AGD severity is a valuable method, particularly on farms as it does not require killing the fish, it can give an overestimate of AGD severity [33]. Lesions that are evident macroscopically can be mistaken as AGD lesions even if they may have another cause, whereas histopathology allows for the association of amoebae with lesions, distinguishing AGD lesions from non-AGD lesions [34]. However, histology reflected gross gill scores in our experiment.

There was some disparity between the presence of amoebae in histology and qPCR results of *N. perurans* numbers, although the results were highly variable irrespective of the method used. There was a greater number of fish identified to have *N. perurans* presence through qPCR compared to histology. Although histology allows confirmation of the direct association of amoebae with lesions on the gills, the processing of samples for histology can result in an underestimation of amoebae numbers [35]. For histological assessment, gills are fixed with formalin or Davidson’s fixative and then processed with ethanol, xylene, and paraffin wax. Although the fixation process provides high quality preservation of cells, the mucus layer is generally lost, and this can result in the loss of associated amoebae [36]. This is more severe with aqueous fixatives such as Davidson’s, compared to non-aqueous fixatives [36]. On top of this, in this study only one gill arch was used, and only a thin section of this arch was sectioned and stained. Although this is a standard practice in histology, as *N. perurans* are a small organism, there is the potential that by taking a small section of the gill, individual amoebae are missed, resulting in an underestimation of amoebae presence. 

Several limitations outside our control may have impacted the ability to fully analyse the efficacy of cold-water treatment. There was high variability in the severity of AGD in fish pre-treatment, for both gross gill score and presence of amoebae on the gills in qPCR analysis and histology. This may have occurred due to the large variation in size of fish used in this study. A recent study has shown that AGD severity has a correlation with fish size, with larger fish being more resistant to severe infections [37]. Regardless of size, there may have been reduced severity of AGD as a result of selective breeding of AGD resistant Atlantic salmon in Tasmania, which have increased variation in the susceptibility to AGD in the population. 

There was also low availability of *N. perurans* for the challenge over the course of this study, which resulted in multiple infection rounds being required to ensure a high enough level of infection to test the experimental treatments. This may have contributed to the variability of infection between fish. However, this method may better reflect the way that fish are infected on farms, resulting in a more realistic depiction of AGD severity across a population. AGD infections on commercial farms are spread between fish over time, likely resulting in a gradual exposure to *N. perurans*, rather than infection with a large quantity of amoebae at one time. 

This study used a conservative time of 15 min to examine the efficacy of cold-water bath treatments; however, based on previous cold-water baths examined for the treatment of sea lice in Atlantic salmon, longer treatments up to 30 min may be possible with no negative effect on the salmon [10]. Temperatures higher than 3 °C could also be investigated. Research into the effects of exposure of Atlantic salmon in low temperatures for varying time periods may be useful to understand the potential maximum duration of cold-water treatments. Due to physiological differences, particularly in temperature tolerance of Atlantic salmon farmed in areas colder than Tasmania, cold-water treatment of AGD maybe more promising in other salmon farming regions. 

In this study, *N. perurans* DNA was detected even on fish treated with the 2 h freshwater bath, where the majority of amoebae would be expected to have been removed from the gills. However, it should be noted that qPCR analysis is not able to distinguish between viable and non-viable amoebae. It may be useful for future studies of AGD to develop molecular methods differentiating viable and non-viable amoebae.

Further development of management practices to reduce AGD in aquaculture is necessary, for both prevention and treatment of the disease. Host behaviour has been explored in parasite prevention, particularly for sea lice in Atlantic salmon. Using behaviour as an early indicator of infection or to promote disease resistance and manipulating behaviour of host fish to promote welfare have been suggested, and would be useful to explore further for management of AGD [38]. The use of snorkel cages for example, utilises fish behaviour by providing a layer of freshwater at the top of pens that salmon can swim through when surfacing to refill their swim bladders, and has been suggested as a method of treating AGD, as well as sea lice, through frequent exposures [23]. Experimental results showed repeated exposures of greater than 3 min to freshwater consistently caused amoebae to detach from gills and caused shedding of mucus and debris from gills [23]. 

Current freshwater bathing involves transfer of fish into well boats that contain large tanks of freshwater for treatment that can be controlled at certain temperatures if required. However, this process can take 1−2 h for the large number of fish produced on farms to be loaded into the tanks, which makes treatment with cold water impossible using this method, as the first fish to be transferred would be exposed to low temperatures for too long. If further study showed potential for cold-water treatment, new infrastructure would need to be developed for shorter cold-water treatments. 

## 5. Conclusions

We demonstrated that In vitro short-term exposures of 15 min and 2 h to cold water caused increased detachment of *N. perurans* to their substrate but did not have an effect on survival of the amoebae. However, In vitro results showed that exposure to 3 °C was most effective when combined with freshwater, but only more effective than 15 °C freshwater at 2 h. Subsequently, 15 min cold-water treatment of AGD affected Atlantic salmon in freshwater or seawater was not more effective at treating AGD than commercial 2 h freshwater bath. Although cold-water treatment may have some potential in management of AGD, the timing of the treatment, temperature, and logistics need to be further investigated.

## Data Availability

The data presented in this study are available on request from the corresponding author.
